# Population Screening for Hereditary Haemochromatosis—Should It Be Carried Out, and If So, How?

**DOI:** 10.3390/genes15080967

**Published:** 2024-07-23

**Authors:** Martin B. Delatycki, Katrina J. Allen

**Affiliations:** 1 Victorian Clinical Genetics Services, Parkville, VIC 3052, Australia; 2 Murdoch Children’s Research Institute, Parkville, VIC 3052, Australia; katieallenk3142@gmail.com

**Keywords:** haemochromatosis, screening, health economics, penetrance

## Abstract

The Human Genome Project, completed in 2003, heralded a new era in precision medicine. Somewhat tempering the excitement of the elucidation of the human genome is the emerging recognition that there are fewer single gene disorders than first anticipated, with most diseases predicted to be polygenic or at least gene-environment modified. Hereditary haemochromatosis (HH) is an inherited iron overload disorder, for which the vast majority of affected individuals (>90%) have homozygosity for a single pathogenic variant in the *HFE* gene, resulting in p.Cys282Tyr. Further, there is significant benefit to an individual in identifying the genetic risk of HH, since the condition evolves over decades, and the opportunity to intervene and prevent disease is both simple and highly effective through regular venesection. Add to that the immediate benefit to society of an increased pool of ready blood donors (blood obtained from HH venesections can generally be used for donation), and the case for population screening to identify those genetically at risk for HH becomes more cogent. Concerns about genetic discrimination, creating a cohort of “worried well”, antipathy to acting on medical advice to undertake preventive venesection or simply not understanding the genetic risk of the condition adequately have all been allayed by a number of investigations. So why then has HH population genetic screening not been routinely implemented anywhere in the world? The answer is complex, but in this article we explore the pros and cons of screening for HH and the different views regarding whether it should be phenotypic (screening for iron overload by serum ferritin and/or transferrin saturation) or genotypic (testing for HFE p.Cys282Tyr). We argue that now is the time to give this poster child for population genetic screening the due consideration required to benefit the millions of individuals at risk of *HFE*-related iron overload.

## 1. Introduction

Hereditary haemochromatosis (HH) refers to a group of genetic iron overload disorders. Homozygosity for the HFE p.Cys282Tyr substitution is by far the most common genetic cause of HH [1]. Untreated, HH can result in multisystem morbidity and mortality, in particular that related to hepatic cirrhosis and carcinoma, diabetes mellitus and, less commonly, cardiac and pituitary morbidity. It can also result in symptoms that present in non-specific ways to a myriad of health care providers, such as arthritis and fatigue [2]. If iron indices are maintained in the normal range, life-threatening morbidity can be avoided [3]. This is most simply achieved by venesections to normalise serum ferritin (SF). Blood obtained from an individual being treated for HH iron overload can be safely used for donation, subject to the same safety assessments as for any blood donor [4]. The American College of Medical Genetics and Genomics (ACMG) have published a list of 81 genes in which pathogenic variants result in “actionable morbidity” [5]. The list includes *HFE*, in recognition that it is a disease that is easy to prevent through regular venesections.

There has been considerable debate as to the merits of community screening for HH and, if introduced, how this should be carried out. Here, we will outline what is known about the impact of *HFE*-related HH, possible models of screening, and the arguments for and against introducing screening in populations with a high prevalence of this condition.

## 2. How Common Are Pathogenic Variants in *HFE*?

There are two common pathogenic variants in HFE, p.Cys282Tyr and p.His63Asp. The p.Cys282Tyr pathogenic variant is present in a heterozygous state in around 1 in 10 northern Europeans, whilst p.His63Asp is present in around 1 in 5. Approximately 1 in 200 northern Europeans is homozygous for p.Cys282Tyr, whilst around 1 in 100 are compound heterozygous for p.Cys282Tyr/p.His63Asp [6,7]. The frequency of these *HFE* genotypes is lower in southern Europeans and lower still in those with ancestry from outside Europe.

## 3. How Is HH Diagnosed?

Those with iron overload will generally have raised serum ferritin (SF) and transferrin saturation (SF). The SF level is correlated with the degree of iron overload [3]. Severe morbidity, including liver cirrhosis, is rare in those with SF less than 1000 µg/L [8,9]. Where iron studies reveal raised SF and TS, the next recommended test is *HFE* genotyping for p.Cys282Tyr and p.His63Asp [10]. If the individual has biallelic variants in this gene, a diagnosis of iron overload due to HH can be made. Various investigations are then recommended to assess the degree of morbidity, including liver imaging and biopsy when the SF is greater than 1000 µg/L [11,12]. Testing for diabetes mellitus should also be carried out. 

The degree of iron overload can be assessed by liver biopsy, liver imaging (ferriscan) and quantitative phlebotomy (the number of units of blood that are removed to normalise SF) [3]. The impact of severe iron overload on the liver can be assessed by liver biopsy as well as non-invasive imaging (transient elastography) and various algorithms based on blood parameters, such as Hepascore, APRI and Fib4 [13]. Regular imaging and α fetoprotein measurement is recommended for monitoring for hepatocellular carcinoma in those with cirrhosis due to HH [12].

## 4. What Is the Evidence for Morbidity from *HFE* Pathogenic Variants?

Morbidity is far higher in those homozygous for p.Cys282Tyr than those compound heterozygous for p.Cys282Tyr/p.His63Asp [6,7,8]. Several studies have examined the penetrance of morbidity in those with these genotypes and the frequency of morbidity in different parts of the world.

An early study by Beutler and colleagues suggested very low penetrance of p.Cys282Tyr homozygosity in the US [14]; however, the methodology of this study was criticised [15].

The Healthiron study identified 203 p.Cys282Tyr homozygotes in an Australian community cohort of 31,192 [8]. The minimum penetrance of disease was found to be 28% for males and 1% for women.

The HEIRS study identified 299 p.Cys282Tyr homozygotes in a community cohort of around 100,000 [6]. There were increased rates of fatigue and arthritis in this group but, rather surprisingly, no increase in liver disease, heart disease or diabetes [16].

More recently, a very large and well-conducted population study based on the UK Biobank identified 2890 p.Cys282Tyr homozygotes among over 450,000 individuals [7]. Male homozygotes had significantly higher rates of liver disease, diabetes mellitus and arthritis than those wildtype for this pathogenic variant. Almost 22% of men and 10% of women were diagnosed with HH by the end of follow-up. This study identified excess mortality in males and females homozygous for HFE p.Cys282Tyr [17]. As the largest study to date, it provides solid population-based evidence that HFE p.Cys282Tyr homozygosity significantly increases the risk of morbidity and mortality if left undiagnosed and untreated.

## 5. How Is HH Managed?

In general, people with a proven iron overload are managed with regular venesection [2], although erythrocyapheresis (the removal of red blood cells with the return of plasma to the donor) can remove iron at a faster rate [18]. Once SF is normalised, the frequency of iron reduction therapy is reduced. Iron chelation therapy can be used in those in whom venesection is not tolerated, such as people with anaemia [12].

## 6. What Is the Evidence for the Benefit of Early Intervention to Prevent Morbidity from Iron Overload?

There is clear evidence for the benefit of the normalisation of body iron in those with HH and iron overload. Individuals homozygous for HFE p.Cys282Tyr without cirrhosis, who have body iron normalised, have reduced morbidity and mortality compared to those with a marked iron overload [19]. Hepatic fibrosis can be reduced by iron depletion and the risk of hepatocellular carcinoma is reduced [20]. The impact of iron reduction for established cirrhosis is less significant and arthritis may or may not respond to body iron normalisation [2,3].

There has been one randomised, patient-blinded trial of iron normalisation in HH [21]. This trial examined patient reported outcomes and biomarkers in HFE p.Cys282YTyr homozygotes with a moderate iron overload (SF between 300 µg/L and 1000 µg/L), randomised to the normalisation of SF by erythrocytapheresis or sham treatment through plasmapheresis. Those whose SF was normalised reported significantly greater improvement in the primary outcome measure, the modified fatigue impact scale, than those whose SF was not normalised, and therefore the study authors concluded that iron normalisation should be instituted in all those with evidence of iron overload and not only those with SF levels greater than 1000 µg/L.

## 7. Genotypic or Phenotypic Screening?

There are two ways people in the community can be identified as being at risk of morbidity from *HFE*-related HH; through genotypic or phenotypic screening. Genotypic screening generally refers to testing people for HFE p.Cys282Tyr with or without p.His63Asp. Phenotypic screening is conducted by measurement of SF and/or TS. The pros and cons of each as a method of screening for HH are outlined in Table 1.

## 8. What Is the Psychosocial Impact for People Identified as Being at Risk of Morbidity from HH through Community Screening?

Several studies have examined the psychosocial outcome for genotypic *HFE* screening. A study of around 11,000 people screened for p.Cys282Tyr in the workplace found p.Cys282Tyr homozygotes had no significant change in anxiety nor health perception from pre-test to post-result [22]. Similar results were obtained in a study of almost 6000 senior high school students screened for the same variant [23]. In the HEIRS study, there were also minimal negative psychological impacts identified from the genetic screening for HH [24].

## 9. What Is Known about the Economic Impact of Screening for HH?

There have been a number of health economic analyses of screening for HH, with most demonstrating cost savings from screening compared to the status quo [25,26,27,28]. This has been found to be true for screening by both genetic testing and for raised iron indices. Some studies identified phenotypic screening to be the more cost effective, whilst others found the reverse to be the case [28].

## 10. Models for Possible Screening Programmes

Community screening for HH could be through a stand-alone programme or could be incorporated into other screening programmes. A stand-alone programme has the advantages that it is specific to HH, it reaches the most eligible individuals, and people can make a specific decision as to whether or not to have such a screening. Research has shown that such a screening can be carried out in a way that allows people to make informed decisions about screening, and there is minimal harm from such a screening [6,22,23]. The main disadvantage is that there are considerable costs to set up the programme, and to obtain and test samples.

Adding HH screening on to other testing is less costly than a stand-alone programme; however, it is only available to people who have undergone genomic sequencing for another reason. Increasing numbers of people are undergoing genomic sequencing, such as whole exome or whole genome sequencing, for a variety of reasons. The data generated for this testing can be assessed for the presence of HFE p.Cys282Tyr with our without p.His63Asp. Examples of where this opportunity arises include people undergoing genomic sequencing to look for the cause of a phenotype in the individual, a parent undergoing sequencing to interpret the result of testing in their child (for example trio genomic testing) or testing for the purpose of reproductive carrier screening [29]. The relative incremental cost to assess the *HFE* gene for one or both variants is low, and there are data to indicate that many people value the ability to have genomic data analysed for so-called secondary actionable variants [30].

The American College of Medical Genetics and Genomics (ACMG) have published a list of 81 genes in which pathogenic variants result in actionable morbidity [5]. The term “actionable” means that people with such a variant(s) can take steps to prevent morbidity. *HFE* is one of these genes, although it is notable that this gene was absent from the initial list of 56 genes proposed [31]. The ACMG called for laboratories to routinely analyse these genes in individuals undergoing genomic sequencing. Whilst a majority of surveyed laboratories in the US routinely examine these additional genes [32], in 2015, only 4/24 laboratories in Europe and Canada and none of the six Australasian laboratories questioned did so [33].

## 11. What Do Guidelines and Commentators Recommend in Relation to Community Screening for HH?

Publications soon after the discovery of pathogenic variants in *HFE* as the most common cause of HH largely voiced opposition to the introduction of community screening for HH. This was based on concerns about disease penetrance, the optimal management of those found to be at risk, and in relation to psychosocial impacts [34]. Similarly, several guidelines have recommended against screening [12,35]. A recent review article suggested the screening of young Caucasian males for HFE p.Cys282Tyr as this has the highest diagnostic yield of those at significant risk of severe morbidity and mortality for the lowest cost [2]. Individuals from support organisations expressed opposition to the limited scope of screening recommended, calling for screening to be available to all and to include testing for HFE p.His63Asp in addition to p.Cys282Tyr [36,37]. The US Preventive Services Task Force produced an opinion on this topic in 2006, recommending against the introduction of screening for haemochromatosis. Their website has an undated statement that “The U.S. Preventive Services Task Force (USPSTF) has decided not to review the evidence and update its recommendations for this topic. The previous evidence review and recommendation may contain information that is outdated.” As noted in the current manuscript, there has been considerable research since 2006 and enormous advances in genetics that has changed the landscape for how screening might be offered. Therefore, the 2006 opinion from the USPSTF cannot be considered as an up-to-date contribution to this debate.

## 12. The Way Forward

Since the identification of the *HFE* gene and its role in HH [1], there has been a very large body of work examining key concerns that were raised in relation to introducing community screening for HH. Most of these concerns have been allayed. Screening can be carried out in a way that people make informed decisions about whether or not to have screening, the screening is cost effective, and there is minimal psychological harm from the process of screening. The penetrance has been shown to be significant and the benefit of preventing body iron reaching levels that predispose to irreversible morbidity and mortality, in particular hepatic cirrhosis and carcinoma, is irrefutable. 

In light of the knowledge that has accrued, we make the following recommendations:

1. Screening should be by genotype and not biochemical testing, as the advantages of the former outweigh the advantages of the latter.
2. Screening should be offered for HFE p.Cys282Tyr and not p.His63Asp, as there are now convincing data that compound heterozygosity for HFE p.Cys282Tyr/p.His63Asp does not lead to sufficient morbidity to warrant inclusion [7].
3. Any adult that undergoes genomic sequencing should be offered the opportunity to know if they have actionable findings, including HFE p.Cys282Tyr.
4. Governments need to recognise the benefits of people being made aware they are at genetic risk of this easily prevented disease, which would prevent negative impact on quality of life, reduce mortality and save money that can be spent on other community needs.

It is time to stop asking why and to start asking why not introduce screening for HFE p.Cys282Tyr in those communities where European ancestry predominates.

## Figures and Tables

**Table 1:** The pros and cons of genotypic and phenotypic screening for HH.

	Pros	Cons
**Genotypic**	1. Needs to be performed once in a lifetime 2. Can be obtained from a broad genomic test such as whole exome or whole genome sequencing 3. Low cost, and becoming lower as the cost of high throughput genetic sequencing continues to decrease 4. At-risk individuals can take steps to prevent disease before symptom onset.	1. Cannot diagnose HH due to other genotypes 2. Cannot diagnose iron deficiency 3. Identified individuals may not be destined to develop the disease 4 Identified individuals may become “worried well” and be overtreated with venesection, resulting in iron deficiency
**Phenotypic**	1. Can diagnose HH due to any genotype 2. Can diagnose iron deficiency	1. Needs to be repeated multiple times in a lifetime 2. High false-positive rate 3. Two-step, so high rate of being lost to follow-up
